# Minimally invasive surgery in Crohn’s disease: state-of-the-art review

**DOI:** 10.3389/fsurg.2023.1216014

**Published:** 2023-07-17

**Authors:** Wei Liu, Wei Zhou

**Affiliations:** Department of General Surgery, Sir Run Run Shaw Hospital, School of Medicine, Zhejiang University, Hangzhou, China

**Keywords:** surgery, laparoscopy, laparoscopic surgery, robotic surgery, transanal surgery, complication, complex

## Abstract

Surgery for Crohn’s disease (CD) has undergone significant advancements over the last two decades, especially minimally invasive surgery. In addition to its feasibility and safety, minimally invasive surgery provides manifold advantages, including a decreased hospitalization duration, improved aesthetic results, and fewer occurrences of intra-abdominal adhesions. Due to the special intraoperative characteristics of CD, such as chronic inflammation, a thickened mesentery, ﬁstulas, abscesses and large masses, a minimally invasive approach seems to be challenging. Complete implementation of this technique for complex disease has yet to be studied. In this review, we provide a review on the applicability of minimally invasive surgery in CD and future perspectives for the technical advances in the field.

## 1. Introduction

Crohn’s disease (CD) is a long-term condition that causes inflammation throughout the layers of the bowel, which recurs periodically, resulting in a chronic state of the disease and leading to various complications, such as thickening of the intestinal walls, formation of abscesses and fistulas, development of strictures, and even perforation. Although medical management has made significant advances, a considerable portion of patients still suffer from disease progression. Fifty to eighty percent of CD patients will require bowel resection in their lifetime (1). Furthermore, CD is a recurring disease, and many patients will require further operation (2). The past few decades have witnessed a major breakthrough in surgical procedures with the introduction of minimally invasive techniques, leading to reduced hospital stays, lower complication rates, better cosmetic outcomes and fewer intra-abdominal adhesions (3). However, because of unfavourable CD characteristics such as chronic inflammation, thickened mesentery, ﬁstulas, abscesses and large masses, a minimally invasive approach seems to be challenging. This review provides an overview of the current state of minimally invasive surgical treatments for CD (Table 1), aiming to provide an evidence-based assessment and recommendations.

**Table 1:** Summary of minimally invasive surgery in CD.

Procedures	Description	Advantages	Disadvantages	Application
Conventional laparoscopic surgery	Use of multiple laparoscopic ports and an extraction site	Faster recovery of bowel function, decreased hospitalization duration, lower incidence of complications, reduced risk of mortality	Moderately steep learning curve	Most widely used procedure; both simply and complex CD
Hand assisted laparoscopic surgery (HALS)	Use laparoscopic tools with one hand, the surgeon inserts other hand through a Gelport device into the abdomen	More accessible to surgeons who have limited experience	Increased equipment cost; limited view and access to the surgical site	Broadly applicable
Single incision laparoscopic surgery (SILS)	A device with multiple ports is inserted through the extraction incision	Decreased pain, hernia, and adhesions	Hinder triangulation and has a steep learning curve	Mainly for ileocolonic resection
Robotic surgery	Use of robotic systems to assist the surgeon in performing surgical procedures	Improved visualization, greater control of the surgical field, and increased dexterity	Rise in cost and a challenging learning curve	Complex and constricted anatomical structures such as the pelvis
Transanal surgery	Involves accessing the surgical site through the anus	No external incision; provides enhanced visualization of the lower pelvis	Limited applicability, steep learning curve	Inflammatory conditions affecting the rectum

## 2. Search strategy

A comprehensive literature review was conducted utilizing the PubMed and Web of Science databases to identify scholarly articles pertaining to the realm of minimally invasive surgery for CD. The search strategy employed the following search terms: “Crohn disease” or “inflammatory bowel diseases” in conjunction with “surgery,” “laparoscopy,” “laparoscopic surgery,” “robotic surgery,” “transanal surgery,” “complication,” “complex,” “fistulizing,” “stricturing,” “recurrent,” or “penetrating.” Each study retrieved underwent thorough evaluation, and the most pertinent articles pertaining to each specific topic were subjected to in-depth analysis and subsequent discussion.

## 3. Minimally invasive techniques

### 3.1. Conventional laparoscopic surgery

Laparoscopic surgery for CD was first described by Milsom et al. in 1993 (4). Since then, the use of laparoscopy has gradually increased in patients with CD. Conventional laparoscopic surgery (laparoscopic-assisted surgery) typically involves the use of multiple laparoscopic ports with diameters ranging from 5 to 12 mm, as well as an extraction site measuring approximately 3–5 cm for specimen removal, resection, and anastomosis. It is the most widely used procedure with the benefits of a shorter time to oral intake, faster recovery of bowel function, decreased hospitalization duration, lower incidence of perioperative complications, lower rates of incisional hernias and adhesions, and improved cosmetic appearance, with CD recurrence comparable to open surgery (5). The operative time and blood loss were similar when compared laparoscopic vs. open surgery in patients with previous laparotomy in a case-matched study (6). Many studies have assessed laparoscopic procedures for CD, confirming feasibility and safety not only for simple cases but also for those patients with recurrent disease and complex fistulizing disease (7–9). The ECCO guideline stated, “Laparoscopic surgery should be offered as the first-line approach in surgery for Crohn’s disease, dependent on appropriate expertise” (10).

There are some difficulties during the laparoscopic procedure for CD, such as severe adhesion, fistula, abdominal abscesses, inﬂammatory masses, and thick and friable mesentery. The absence of tactile feedback may also pose limitations in identifying the anatomy during the procedure. Ileocolic resection for simple stricturing disease may be suitable for beginners. Even with the growing experience, the conversion rate to open surgery remains considerable. Mege et al. described 458 laparoscopic procedures performed in 427 patients and found that the laparoscopy rates increased over time, but 20% of selected cases still required conversion to open surgery. Recurrent disease, thickened mesentery, a large inflammatory mass, and extensive disease were among the factors identified as predictive of the need for conversion (11). A recent study investigated the indications for opting for an upfront open approach in ileocolic resection for CD. The researchers identified that the involvement of the abdominal wall or the presence of concomitant open procedure, or anesthesiologic contraindication to MIS serves as a no-go for the MIS approach (12).

In a randomized controlled trial, laparoscopic-assisted ileocolic resection was compared to open ileocolic resection for primary CD (13). The conversion rate was found to be 10%. Laparoscopic surgery had a median operating time of 25 min longer than open surgery but resulted in significantly lower morbidity, hospital stay, and overall costs. The authors concluded that laparoscopy was the preferred approach for the treatment of distal ileitis in CD. It is crucial to remember that the patients’ conditions should be optimized before surgery while handling CD patients with complicated diseases. Abdominal abscesses should be drained percutaneously. Nutritional optimization should be performed. Bowel maps displayed by CT or MRI provide a comprehensive and objective assessment of the disease. These management strategies decrease the rate of conversion and postoperative morbidity (14).

### 3.2. Hand-assisted laparoscopic surgery (HALS)

The hand-assisted laparoscopic procedure combines the benefits of minimally invasive surgery with the potential for tactile input and manual help, enabling increased visibility of the anatomy and better access to the bowels. While using laparoscopic tools with the other hand, the surgeon inserts one hand through a Gelport device into the abdomen. As a useful substitute for traditional laparoscopic surgery, it is simpler for surgeons with little prior familiarity with the procedures. Thirty-eight consecutive patients who underwent subtotal or complete colectomy were studied by Nakajima et al. The median duration of the operation was significantly shorter in HALS (251 min) than in laparoscopic surgery (330 min). There was no significant difference observed in postoperative complications between the groups (15). However, with the wider use of laparoscopic procedures and improving learning curves in minimally invasive surgery, the number of HALS procedures have decreased. For more complex cases, such as fistulizing disease and extensive Crohn’s colitis, HALS has the potential to decrease the operative time while still maintaining its less invasive nature.

### 3.3. Single incision laparoscopic surgery (SILS)

Single-incision laparoscopic surgery is a subtype of laparoscopic surgery that uses a single incision to minimize all ports to one site. Laparoscopic instruments are introduced from a single paraumbilical or transumbilical incision. This approach enables the entire procedure to be conducted through a single point of entry, which can also serve as the site for extracting the specimen. In a propensity score-matched analysis, 174 patients who received ileocolonic resection were divided into SILS, laparoscopy and open surgery groups (16). The conversion rate was found to be 10.3% for SILS and 12% for traditional laparoscopy, with no statistically significant difference in the postoperative complication rates between the two techniques. In a multicentre study, in comparison to standard laparoscopic surgery, SILS had a similar operative time, might be less painful and patients might require less opioid analgesia (17). The length of hospital stay in the SILS group was significantly shorter (5 days) compared to 7 days for laparoscopy and 9 days for open surgery. Furthermore, a few studies have analysed the feasibility of single-port laparoscopy in patients with complicated (fistula, abdominal abscess, or mass) or recurrent disease (17, 18). Although the conversion rate to open surgery tended to be higher, the postoperative morbidity was similar, concluding that SILS is feasible and safe, even for complex CD. However, SILS is likely to be a more challenging technique than traditional laparoscopy, as it may limit the surgeon’s visibility and mobility and has a steep learning curve.

### 3.4. Robotic surgery

The use of a robotic surgical platform allows for improved visualization, greater control of the surgical field, and increased dexterity for the surgeon. Better viewing contributes to removing adhesions or resection of diseased bowel without damaging adjacent organs. In addition, the application of a robotic approach can potentially address the ergonomic and visual limitations of laparoscopic surgery, particularly in the context of complex disease and a narrow space, such as in the pelvis. Furthermore, when performing procedures such as intramesorectal or total mesorectal excision, the use of robotic technology may aid in the preservation of nearby nerves along the surgical plane (19). It is important to note that a single docking for robotic surgery is typically inadequate, and multiple dockings may potentially prolong the surgical time. To date, there have been only a few small case series reporting the use of robotic surgery for CD. Aydinli et al. reported robotic ileocolic resection, with a shorter bowel function time and a longer operative time compared to standard laparoscopy (20). Rencuzogullari et al. showed a longer operative time and higher blood loss in robotic proctectomy in a case-matched comparison with laparoscopic surgery (21). Robotic surgery demands an entirely different skill set and learning curve and signiﬁcantly increases patient costs. With increased experience, surgical proficiency, and ongoing technological advancements, these problems associated with robotic surgery for CD may be mitigated. Consequently, robotic surgery may become a valuable instrument for surgeons to efficiently manage CD-related complications. In certain unique circumstances, a combination of robotic and laparoscopic techniques may simplify complex surgical procedures.

### 3.5. Transanal surgery

The introduction of transanal total mesorectal excision (TaTME) as a surgical approach for managing rectal cancer has offered novel operative strategies that may be applicable to the surgical management of inflammatory conditions affecting the rectum. For patients with rectal stenosis or extensive perianal disease, proctectomy may be indicated. The transanal surgical technique starts with perineal dissection along the intersphincteric plane, after which a transanal port can be inserted into the perineal wound to facilitate bottom-up dissection. Initially, the posterior plane is dissected, creating a working space. After mobilizing the rectum posteriorly and laterally, anterior dissection can then be performed (22). CD surgery does not require anatomic oncologic resection, and close rectal dissection can be used, avoiding injury to the pelvic nerves and internal iliac vessels. The transanal approach provides enhanced visualization of the lower pelvis and can help overcome the difficulties associated with accessing and operating in a narrow, scarred, and fibrotic pelvic area (23).

## 4. The application of minimally invasive techniques

### 4.1. Uncomplicated small bowel and ileocolic disease

Given that the terminal ileum is the most commonly affected site in CD, ileocolic resection is the most frequently performed surgical intervention. In regard to managing stricturing CD that is limited to the terminal ileum, a laparoscopic approach is typically considered the preferred method of treatment. Ileocolic and small bowel resection was the chosen procedure when laparoscopy was first introduced in CD. In the ECCO guidelines, a laparoscopic approach is preferred for ileocolic resections (10).

It is universally known that CD is commonly associated with a progressive course; for patients who do not respond to conventional treatments, early anti­TNF therapy is the preferred choice. In a randomized controlled, open-label trial of LIR! C trial, patients with nonstricturing ileocecal CD (terminal ileum <40 cm) in whom conventional therapy failed were randomly allocated to receive laparoscopic ileocecal resection or infliximab (24). The findings revealed that in patients with limited CD, laparoscopic ileocecal resection was comparable to infliximab therapy with regard to quality of life and was not associated with higher rates of morbidity. Laparoscopic ileocecal resection was associated with significantly lower total CD-related direct health care costs than infliximab treatment (25). In the long-term follow-up, half of the patients who received infliximab required surgery, while the other half still needed biological therapy (26). Nearly half of the patients who underwent ileocecal resection did not need any further medical treatment within 5 years. This study is a landmark in demonstrating the potential advantages and long-term disease control of early laparoscopic ileocecal resection in patients with luminal localized ileocecal CD.

The lateral to median, median to lateral, and retro-mesenteric approaches are the most commonly performed in CD ileocecal dissection, while the latter approach is considered safer because it involves mobilizing the ileo-colon away from the inflammatory site, allowing the caecal region to be addressed later (27). The role of the mesentery has come under close examination in recent times, as it is believed to play a significant part in the pathological mechanisms of CD and potentially in disease recurrence as well (28). In this regard, extended mesenteric resection has garnered considerable attention as a potential therapeutic approach. Inclusion of the mesentery in ileocolic resection for CD has demonstrated a correlation with decreased surgical recurrence rates (29). Laparoscopic resection offers distinct advantages in this context, as it enhances the likelihood of accessing the root of the vessel. It is important to note, however, that CD often presents with severely inflamed, delicate, and thickened mesenteric tissue, necessitating the expertise of skilled surgeons when performing these operations.

### 4.2. Colon resections

Laparoscopic resections for the colon may be challenging due to the broader, thickened mesentery and penetrating disease with ﬁstula and abscess. As experience with laparoscopy has improved, the indications for laparoscopic surgery have now been expanded to complex colonic resections. Umanskiy et al. conducted a study comparing 55 laparoscopic colectomies to 70 open colectomies in patients with CD (30). The study demonstrated that the laparoscopic approach resulted in less blood loss, a faster return of bowel function, and a shorter hospital stay compared to the open surgery group. Another case-matched study was performed to compare short- and long-term outcomes of laparoscopic colectomy with open colectomy in CD (31). The laparoscopic group had a longer operative time, a shorter median length of stay and comparable blood loss and postoperative complications. These findings confirm that laparoscopic colectomy is a safe and effective approach for appropriately selected cases when performed by experienced surgeons. The conversion in laparoscopic colectomy might be higher than that for ileocecal resection. The occurrence of an intraperitoneal abscess or fistula was found to be a significant factor predicting the conversion of laparoscopic colon resections for CD.

### 4.3. Complex CD

CD is a transmural inflammation that can result in penetrating disease with fistulas or abdominal abscesses. Complex CD cases typically encompass recurrent cases and those with fistulizing disease, which can present as abdominal or pelvic abscesses and complex fistulas. Studies have demonstrated that over a 10-year follow-up period, approximately 70% of CD patients may develop complex disease (32). Complex anatomy and severe intra-abdominal inflammation might make the laparoscopic approach challenging. In a case-match study, 11 patients presenting with 13 complex fistulas were matched with 22 simple cases (33). There were no significant differences between the groups in terms of operative time, conversion, or postoperative morbidity. A recent large case series analysed 386 patients who underwent surgery for complex CD, with 193 patients in each group of open and laparoscopic surgeries (34). Laparoscopic surgery was associated with reduced operative times and length of stay. Mortality and the reoperation and symptomatic hernia rates were comparable to those of open surgery. These data showed that the complexity of the disease was not necessarily a contraindication for laparoscopy. Conversion to open surgery was predicted by several factors, including severe adhesions, extensive inflammation or disease involvement, large size of the inflammatory mass, inability to dissect a fistula, or difficulty in assessing the anatomy during laparoscopic surgery (35). Maggiori et al. reported a significant increase in the rate of laparoscopically managed complex procedures over a 14-year period, from 16% to 33%, a decrease in the rate of conversion to open surgery from 18% to 6%, and a decrease in the rate of severe postoperative morbidity from 14% to 8% (36).

Due to the relapse of disease, 40%–50% of CD patients undergoing surgery are likely to need further operations within 10–15 years (37). Several studies have investigated the role of laparoscopic surgery for recurrent CD. Hasegawa et al. compared the outcome of laparoscopic surgery for recurrent and primary disease (38). Although the operating time was longer for the recurrent CD group, there were no significant differences in the rate of postoperative complications. When compared with open repeat surgery, the operative times were similar, and a significant reduction in wound infection rates was revealed in laparoscopic surgery. In a meta-analysis of 627 participants (413 with primary CD and 214 with recurrent CD), recurrent CD had a significantly higher conversion rate (OR=2.5), while the total complication rate had no difference (8). According to these data, the ECCO-ESCP stated “where appropriate expertise is available, laparoscopic surgery should be attempted also in more complex cases or recurrent disease” (39).

**Figure 1:**
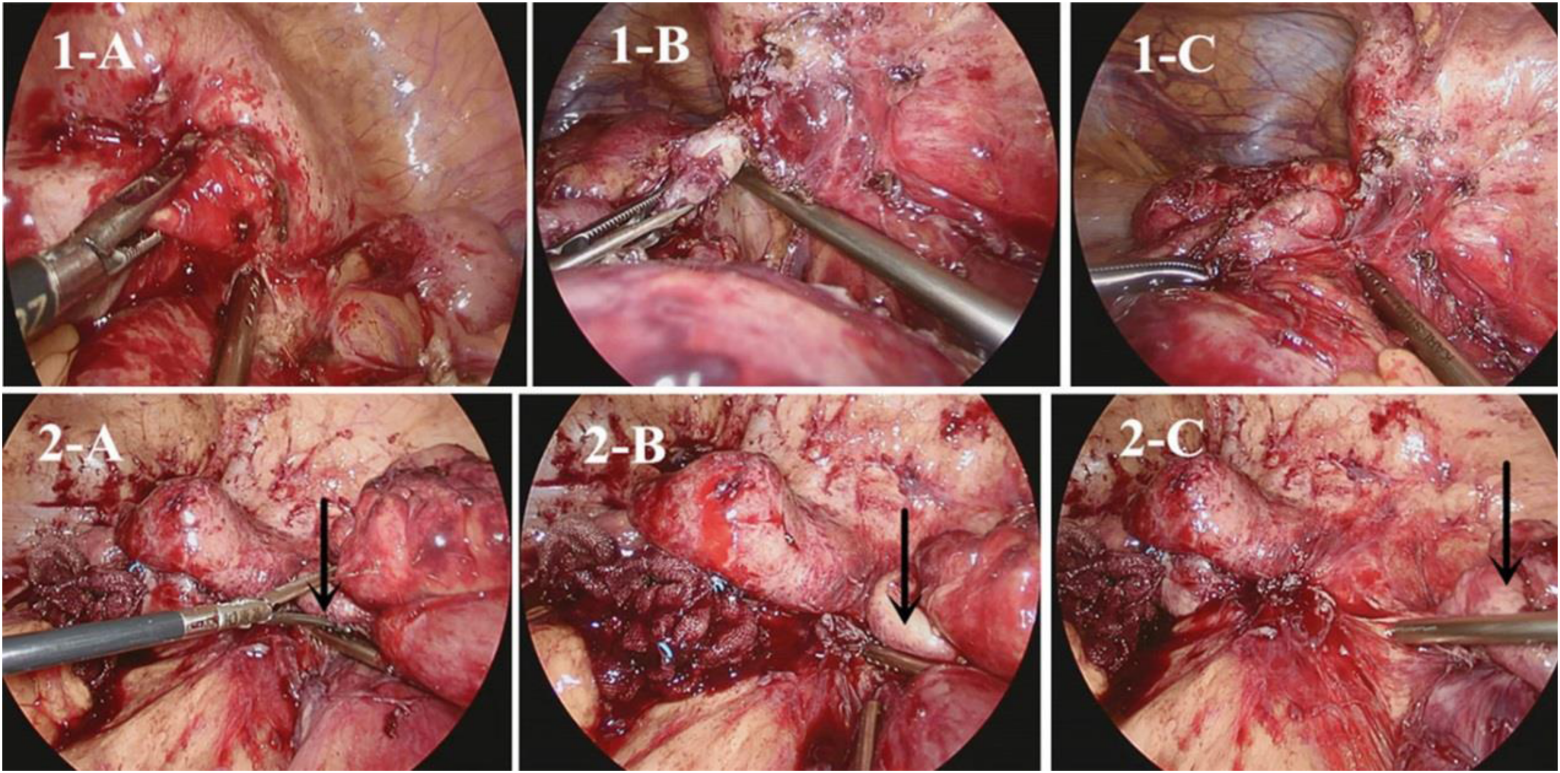
(**1-A**) search for correct tissue layer; (**1**-**B**,**1**-**C**) Use an aspirator to open adhesions (**2**-**A**). Identify the appendix; (**2**-**B**). Perform appendix-directed anatomic dissection; (**2**-**C**). Ensuring complete dissection of the ileocecal region (40).

To address complex CD, we use a laparoscopic suction irrigator technique for blunt separation, together with an ultrasonic knife to free the bowel and mesentery sharply (40). When dealing with abscesses and phlegmon in the ileocecal region, we typically look for the appendix first and use it as a guide for separation (Figure 1). This approach provides clear exposure and reduces the risk of damage to surrounding structures, such as blood vessels and ureters. Dissection usually follows an easy-to-difficult approach. A hybrid laparoscopic/open approach is helpful in certain circumstances, trying as much as possible to reduce the size of the incision. However, in cases of massive bleeding, poor visualization, or injury to other organs, intermediate openings should be performed.

### 4.4. Strictureplasty

Strictureplasty is a recognized and safe surgical approach for small-bowel strictures of CD (41). Sampietro et al. performed strictureplasty by laparoscopy and showed that it was both safe and feasible, resulting in a low rate of complications (42). Tou et al. reported the utilization of robotic technology in strictureplasty, but further investigation is necessary to evaluate the efficacy of this method (43).

**Figure 2:**
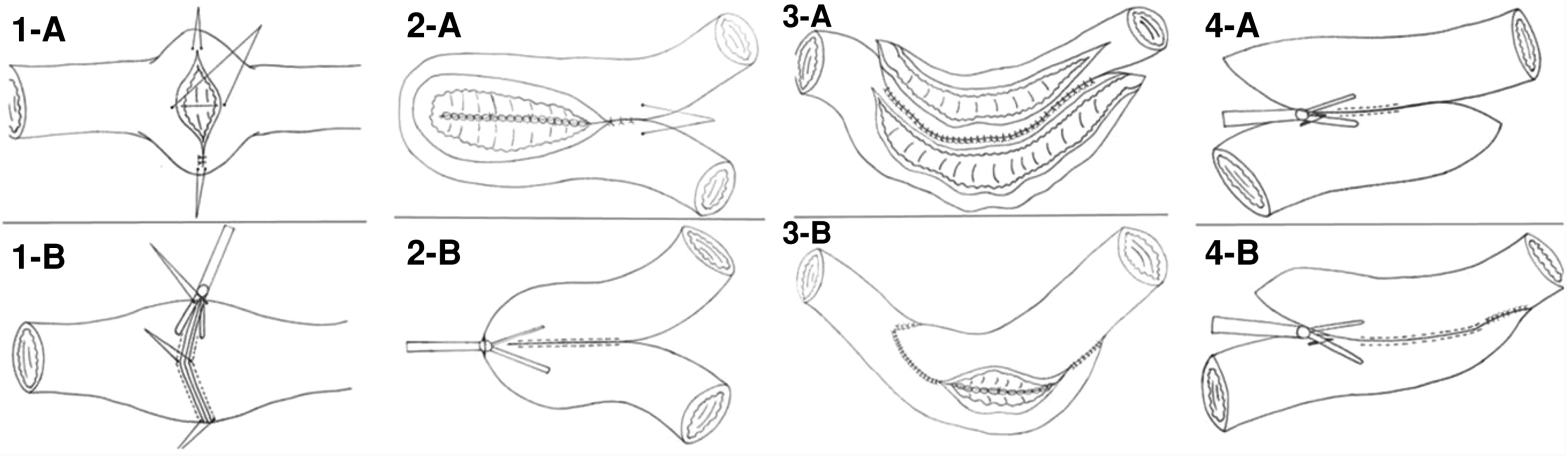
Strictureplasty by handsewm and endoscopic GIA. (**1**-**A**) Handsewm H-M strictureplasty; (**1**-**B**) Stapled H-M strictureplasty; (**2**-**A**) Handsewm Finney strictureplasty; (**2**-**B**) Stapled Finney strictureplasty; (**3**-**A**,**3**-**B**) Handsewm Michelassi stricturoplasty; (**4**-**A**,**4**-**B**) Stapled Michelassi stricturoplasty (44).

Our centre currently utilizes an endoscopic GIA stapler for intestinal strictureplasty, which helps to avoid the need for long enterotomy and extensive suturing (44). These methods can make laparoscopic or robotic strictureplasty easier (Figure 2).

### 4.5. Intracorporeal anastomosis and natural orifice specimen extraction (NOSE)

Intracorporeal anastomosis (ICA) has the benefit of reduced postoperative complications, earlier return to bowel function and better cosmesis. Simultaneously, ICA reduces the risk of tension on the shortened and thickened mesentery and minimizes the length of the extraction site. Bergamaschi et al. reported 80 ileocolic resections by a total laparoscopic intracorporeal procedure (45). The conversion rate was 1.2%, the complication/reoperation rate was 7.5%, and the readmission rate was 3.7%. Their results demonstrated that performing laparoscopic ileocolic resection with intracorporeal vascular division and anastomosis led to a favourable outcome. In a recently published retrospective study, the short-term postoperative results of robotic ileocolic resection for CD in patients who underwent ICA vs. those who received extracorporeal intestinal anastomosis (ECA) were compared (46). The ICA group had a faster return to bowel function (1.6 d vs. 2.1 d), a longer operative time (235 min vs. 172 min) and a comparable postoperative complication rate (22.2% vs. 26.9%). Given that mobilization, devascularization, transection, and anastomosis can all be accomplished laparoscopically, the only necessary reason for enlarging a port incision would be to facilitate the removal of the surgical specimen. Eshuis et al. demonstrated that in the absence of a large inflammatory mass, transcolonic removal of the specimen using a colonoscope is a feasible option for patients with ileocolic CD (47). The postoperative recovery, quality of life, and cosmesis were comparable to those observed following standard laparoscopically assisted resection.

**Figure 3:**
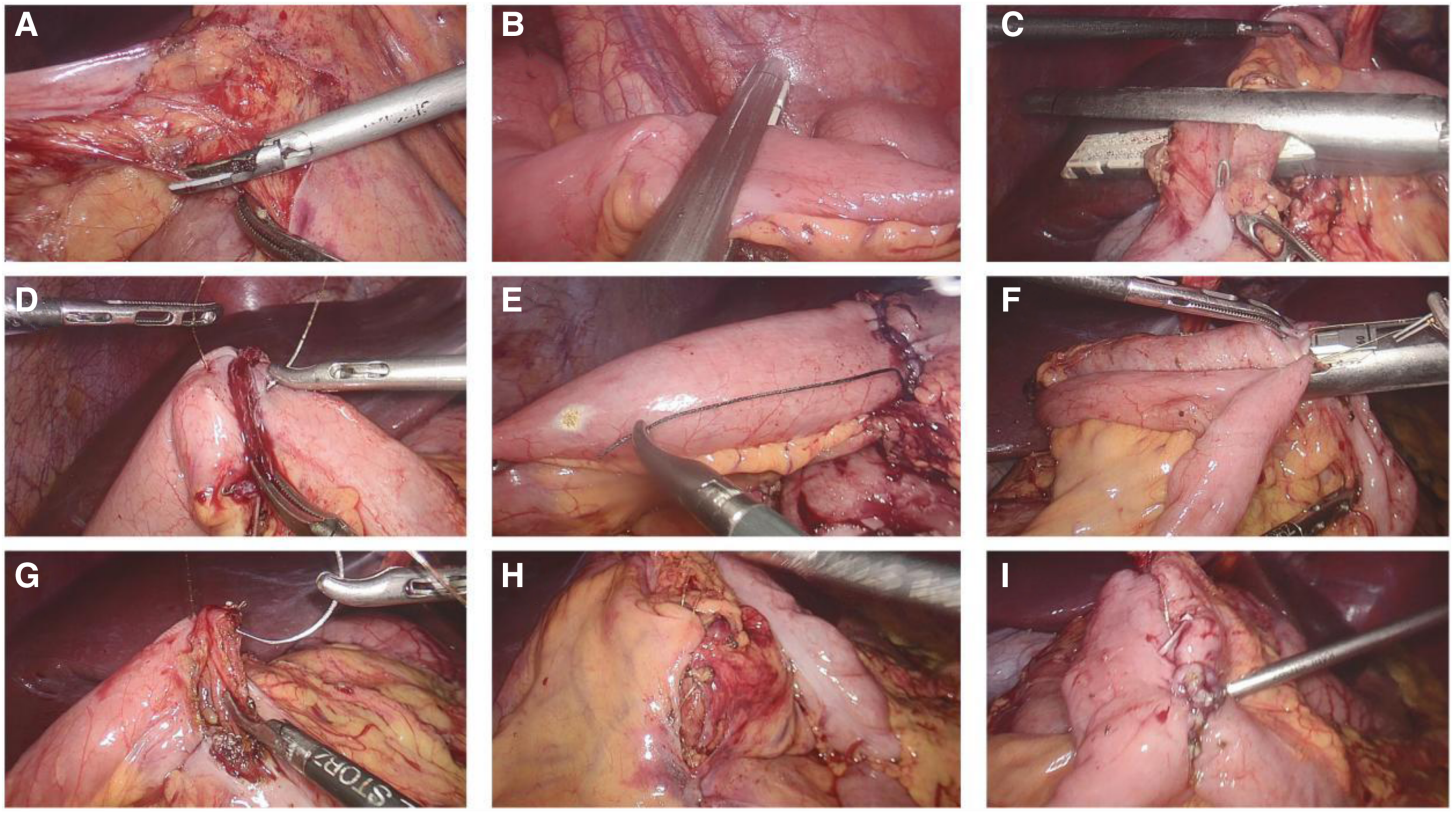
Photographs of the creation of a laparoscopic intracorporeal stapled anastomosis excluding mesentary. (**A**) Divided the mesentery of ileum; (**B**) Transected terminal ileum 2 cm proximal to the diseased bowel, placing the Endo GIA perpendicular to the the mesentery which is located in the middle of the staple lines; (**C**) Transected the colon in a similar manner; (**D**) Sewn two stapled lines together; (**E**) Created an antimesenteric small enterotomy on each stump, 6 cm away from the staple line; (**F**) Fashioned a side-to-side anastomosis with a Endo GIA using one 60 mm-long cartridge; (**G**) Closed the enterotomy; (**H**) Closed the mesenteric defect; (**I**) Morphology of stapled anastomosis excluding mesentary.

A new surgical technique for treating CD is Kono-S anastomosis, which involves creating an antimesenteric functional end-to-end handsewn connection. It has demonstrated highly encouraging outcomes by significantly decreasing the incidence of anastomotic recurrence. Julià et al. conducted a study on a modified Kono-S anastomosis using robotic surgery and concluded that the procedure is both safe and feasible (48). The time to construct the stapled antimesenteric functional end-to-end anastomosis was 21 min (49). While when performed the antemesenteric anastomosis intracorporeally by robotic surgery, anastomosis time was 120 min (48), suggested that intracorporeal anastomosis requires a steep learning curve. The significant reduction in the recurrence rates achieved through the Kono-S anastomosis technique may be attributed to several factors (50). One of these is the “supportive column,” which adds stabilization to the anastomosis and limits luminal distortion. Additionally, the technique isolates the mesentery from the anastomotic lumen. To make it easier to perform laparoscopically, we propose an alternative anastomosis based on Kono-s anastomosis, called stapled anastomosis excluding the mesentery. The procedure is shown in Figure 3. Stapled anastomosis excluding the mesentery seems to be safe and can be easily performed intracorporeally. Future studies are needed to confirm its role in preventing postoperative recurrence.

## 5. Conclusion

Despite the difficulties posed by the surgical management of CD, minimally invasive surgery has become an increasingly popular option. The emergence of CD surgery as a specialized field, with surgeons who focus on the surgical management of inflammatory bowel disease, has helped to establish the optimal role of minimally invasive surgery in treating CD. Given the complex characteristics of CD surgery, the laparoscopic approach should be carefully utilized by highly experienced surgeons. A progressive approach to the surgical management of CD can be taken by beginning with less complex cases, such as short stricturing ileal CD, then proceeding to more challenging cases, such as colon resection, and ultimately addressing advanced forms of CD, such as recurrent or penetrating disease. The decision to adopt which procedure of minimally invasive surgery should be made on a case-by-case basis, and it should depend on the surgeon’s expertise and the patient’s characteristics.
